# Traditional Use of Medicinal Plants in South-Eastern Serbia (Pčinja District): Ethnopharmacological Investigation on the Current Status and Comparison With Half a Century Old Data

**DOI:** 10.3389/fphar.2020.01020

**Published:** 2020-07-08

**Authors:** Jelena Živković, Milan Ilić, Katarina Šavikin, Gordana Zdunić, Aleksandra Ilić, Dejan Stojković

**Affiliations:** ^1^ Department for Pharmaceutical Research and Development, Institute for Medicinal Plant Research “Dr. Josif Pančić”, Belgrade, Serbia; ^2^ Department of Pharmacognosy, Faculty of Pharmacy Novi Sad, University Business Academy in Novi Sad, Novi Sad, Serbia; ^3^ The Clinical Center of Vojvodina, Center for Pathology and Histology, Novi Sad, Serbia; ^4^ Department of Pathology, Faculty of Medicine, University of Novi Sad, Novi Sad, Serbia; ^5^ Department of Plant Physiology, Institute for Biological Research "Siniša Stanković" - National Institute of Republic of Serbia, University of Belgrade, Belgrade, Serbia

**Keywords:** medicinal plants, ethnobotany, Balkan traditional medicine, ethnopharmcology, gastrointestinal ailments, Serbia

## Abstract

Balkan Peninsula is one of the most important biodiversity centers in Europe. Despite that, the usage of plant species in the traditional medicine of some Balkan regions remained largely unexplored in the past. This study aimed to collect and document data on the traditional use of medicinal plants in Pčinja district in South-Eastern Serbia, which is among the least developed regions in Serbia. Also, comparison with data collected by Dr. Jovan Tucakov, in a book called Herbal therapy was conducted. The survey was carried out using semi-structured interviews and 113 informants were interviewed. Quantitative ethnobotany factors were calculated, allowing us discussing the results. The informants reported data on 86 medicinal plants belonging to 43 families in Pčinja district. *Lamiaceae, Asteraceae*, and *Rosaceae* were the dominant locally used families. The species with the highest number of use reports were *Mentha piperita*, *Matricaria chamomilla*, and *Hypericum perforatum*. Gastrointestinal ailments, respiratory problems and skin diseases were the most frequently reported indications. Usually, the administration was primarily oral followed by topical applications. Leaves were dominantly exploited plant parts and the most frequent preparation form was infusion. Medicinal plants in Pčinja district are mainly used as a mode of primary health care for treating minor health issues. After comparing our results with the ones collected half a century ago by Dr. Jovan Tucakov we can conclude that plant species mentioned in our investigation previously had a much wider spectrum of application.

## Introduction

The use of medicinal plants in Serbia for the treatment of numerous health problems has a long history and the oldest documents about this topic date back from the 14^th^ century (The Hodoch Codex) and the 16^th^ century (The Chilandar Medicinal Codex) (Sarić, 1989). Much of the knowledge on the use of medicinal plants has been collected during the second half of the 20^th^ century by a university professor, Dr. Jovan Tucakov, in a book called Herbal therapy (first time published in 1973). This book represents the essential guide-book in this area. According to this author, the history of the medical culture of people from the Balkan is very interesting and complex since in this part of Europe strong influences of East and West are present. Medicinal literature of Mediterranean and other countries has been mixed and intertwined with the folk medicine of the illiterate warriors and shepherds (Tucakov, 1997). Almost every home in Serbia possesses this book and family members rely on it while practicing self-medication (Stojanović et al., 2017). Today, in Serbia, the simultaneous use of herbal preparations together with conventional drug therapy is very frequent (Samojlik et al., 2013).

During the last decade of the 20^th^ century, the health status of the Serbian population sharply deteriorated. This was due to several causes including the dramatic break up of socialist Yugoslavia, series of wars, international sanctions and a general decline of standards of living (Nagorni-Obradović and Vuković, 2014). According to research conducted in 2015 forty percent of Serbian citizens reported that a long-term illness or health problems are somewhat more common among residents of Southern and Eastern Serbia (Lončar, 2016).

In Serbia, the poverty level is the highest in South-east regions where the economy relies on small farming, poor infrastructures and inadequate public services. As a result of economic failures, the entire region is witnessing decade-long depopulation. According to the census performed in 2002, there were 64 inhabitants per km^2^. However, the census of 2011 has indicated the palpable decrease as there were 45 inhabitants living per km^2^ in Pčinja district. This fact testifies about the potential disappearance of traditional knowledge.

The aim of this paper was to analyze ethnomedicinal usage of medicinal plants in Pčinja district in South-Eastern Serbia using data obtained through the semi-structured interviews of autochthonous population and to compare results with previously published ethnomedicinal studies conducted on the territory of Serbia (Jarić et al., 2007; Šavikin et al., 2013; Zlatković et al., 2014; Jarić et al., 2015). Also, we have compared the obtained results with the data collected by Dr. Jovan Tucakov in the book called Herbal therapy. The purpose of this comparison was to investigate how the usage of medicinal plants has been changing over time. More precisely to determine are there any differences in the application of medicinal plants and can we notice any decrease or diversity of use. Also, comparison with previously published data collected from researchers from surrounding territories in Serbia was performed.

## Material and Methods

### Research Area

The research was conducted in the area of Pčinja district, which extends to the south of the Republic of Serbia covering an area of 3,520 square kilometers (3.98% of the territory of the Republic of Serbia). In the east, it borders with Bulgaria, in the south with North Macedonia and Albania, in the west with Kosovo and Metohija and in the north with the district of Jablanica. The district includes municipalities of Vranje, Bujanovac, Bosilegrad, Preševo, Trgovište, Vladičin Han, and Surdulica (Marković and Stevović, 2016).

Pčinja district is the region in South Serbia which is extremely poor. Population aging and negative demographic trends are present, literacy level is low (especially in women over 65 in rural households), socio-economic development is low and high level of depopulation is present. Absence or low quality of road infrastructures, limited access to social and public services, traditional agricultural production on small households characterize the majority of municipalities, especially rural ones. Although health care is available and free for all citizens regardless of social status, Serbian healthcare system has been severely underfunded for decades and therefore the standard of available healthcare has poor quality. Equipment and facilities were not modernized for many years. On the other hand, human resources strategy was not appropriate for decades and education policy was not coordinated with the needs of health care, so the number of unemployed doctors was constantly increasing. At the same time, there was insufficient number of some specialist (radiologist, anaesthesiologist and cardiac surgeons). Low salaries and high unemployed rates create an incentive for doctors to emigrate (Stošić and Karanović, 2014).

Due to that reason, self-medication is popular among inhabitants, as well as simultaneous use of herbal preparations together with conventional drug therapy. The most common causes for going to the Health Centre general practitioner are respiratory and cardiovascular diseases. Also, there is an increase in the number of patients with neurological diseases and substance abuse (which represent a major health and socio-economic problem) (Mužik and Karajičić, 2014).

### Ethnomedicinal Survey

Data was collected using semi-structured ethnomedicinal interviews during the period March-September 2015. A total of 113 informants were interviewed. No special criteria for the selection of the informants were used. The majority of informants (109) were Serbian nationality, one was Macedonian and three did not declare. The age of informants was between 17 and 74, with an average value of 48 years (**Table 1**) and the number of male and female informants was 30 (27.4%) and 81 (72.6%).

**Table 1:** Demografic features of informants in Pčinja district.

	Pčinja district
Total number of informants	**113**
<20	7
20–40	21
41–60	64
61–80	21
>80	0

Interviews were conducted by researchers of the Institute for medicinal plant research “Dr. Josif Pančić”. They were equipped with appropriate terrain vehicles and with knowledge of common locations known for tradition in collecting medicinal plants since they have rich experience in conducting pharmacognostic and resource field research in Serbia. Also, researchers were familiar with the information provided from small and medium collectors of medicinal plants from various parts of Serbia with whom the Institute has long-term cooperation in medicinal plants trade.

For this study, the informants were selected based on the recommendations of local village leader or by known collectors of medicinal plants. Also, in each village, we look for potential informants in gathering places of elderly people and we asked for information about other potential informants through a snowball sampling. Explanations of the background and the aim of the study were provided to the potential informants before starting the surveys. Only those who claimed to know the plants and their medicinal uses were interviewed.

Interviews were conducted orally with all respondents. When it was possible, ethnomedicinal interviews were combined with guided tours, to the locations where the informants usually collect medicinal plants or to the market places, where the informants identified plant material. Moreover, for the identification, researchers were equipped with herbarium, photos of the medicinal plants as well as the relevant literature - Flora of Serbia (Josifović, 1970–1977; Sarić and Diklić, 1986) and Flora of Europe (Tutin et al., 1964–1980; Tutin et al., 1993).

The informants were asked to list all the plants they use in the treatment of different health issues. In particular, the interview included the following questions: respondent name, sex, age, residence, nationality, profession, local names of the plants they use, plant part(s) used, preparation/administration, and folk medicinal uses. Vouchers were collected and dried for herbarium preparations, while photographs were taken for easier identification with the help of standard literature: Flora of Serbia (Josifović, 1970–1977; Sarić and Diklić, 1986) and Flora of Europe (Tutin et al., 1964–1980; Tutin et al., 1993). Some of the plant species were bought in pharmacies. The identity of these commercial products was confirmed *via* their package leaflet and it was considered correct.

All reported plant species were indicated for the citations of 15 out of 17 ailment categories which were established according to the International Classification of Primary Care accepted by the WHO (Direktoratet for e-helse, 2018): general and unspecified (A), digestive (D), blood, blood-forming organs and immune mechanisms (B), endocrine/metabolic and nutritional (T), psychological (P), neurological (N), eye (F), ear (H), cardiovascular (K), respiratory (R), skin (S), musculoskeletal (L), urological (U), pregnancy, childbearing, family planning (W), female genital (X), and male genital (Y).

All plants cited by informants, even when only mentioned by a sole informant, have been considered. After collecting data, plants were ranked based on the number of times that their uses were mentioned by the participants.

Each time a plant was mentioned as ‘‘used’’ it was considered as one ‘‘use-report’’ (UR). If one informant used a plant to treat more than one disease in the same category, it was considered as a single use-report (Trotter and Logan, 1986).

### Data Analysis

The collected ethnomedicinal data were analyzed to obtain information about frequency and percentage of families, the number of most cited plant species and their uses, the most commonly used plant parts as well as preparation methods. The data collected during the field study was sorted in Microsoft Excel and further evaluated by quantifying the use reports according to previously published literature (Trotter and Logan, 1986). Also, informant consensus factor was calculated according to the equitation: FIC = (Nur-Nt)/(Nur-1), where Nur presents the number of use citations in each ailment category and Nt presents the number of species used (Trotter and Logan, 1986). This means that FIC values would be low (near 0) if plants were chosen randomly or if informants do not exchange information about their use. FIC values would be high (near 1) if there were well-defined selection criteria and/or if informants exchange information about plant usage (Teklehaymanot and Giday, 2007).

The obtained data were statistically analyzed using appropriate tests for the determination of statistical significance (chi-square test of independence with correction of continuity according to Yeats).

We have compared the obtained results with the data collected by Dr. Jovan Tucakov in the book called Herbal therapy. The purpose of this comparison was to investigate how the usage of medicinal plant has been changing over time. Data presented in the book Herbal therapy represent knowledge gained through Dr. Jovan Tucakov’s working lifetime, especially during his field research, compiled with the data from relevant scientific literature available at that time. The area of research was predominantly the territory of the today’s Republic of Serbia, but data relating to other parts of the SFR Yugoslavia, the state that existed at the time, are also presented in the book. Unfortunately, precise methodology has not been described.

Also, in order to compare traditional plant use in investigated district with neighboring areas, four previously published ethnomedicinal studies conducted on the territory of Serbia were considered (Jarić et al., 2007; Šavikin et al., 2013; Zlatković et al., 2014; Jarić et al., 2015), having in mind these studies were performed in the closest proximity of the investigated region.

## Results and Discussion

### Ethnomedicinal Survey

The results obtained during our study are presented in **Table 2** where plants are arranged in alphabetical order of their botanical names. For each plant, the botanical name and family, local names, part(s) used, voucher number, preparation/administration, folk medical uses and total number of use reports were reported. In the same table, we have provided data from Herbal therapy written by Dr. Jovan Tucakov that we have used for comparison.

**Table 2:** Plant species used in modern and traditional medicine of Pčinja district.

Scientific name, local Serbian name, family name, voucher number	Part(s) used	Medicinal use^a^	Preparation form	Medical use according to Tucakov (Tucakov, 1997)	Number of use reports
*Achillea millefolium* L., hajdučka trava, sporiš, hajdučica, *Asteraceae*, IPLB36/13	Herb	D: 8 (I: loss of appetite, stomach disorders, liver problems) R: 4 (I: cough and cold) U: 1 (I: renal complaints) K: 2 (I: circulation improvement, against hemorrhoids and palpitations) S: 1 (E: against wounds)	Infusion, Tincture	D: tonic, stomachic, antispasmodic, excessive intestinal gas and flatulence, chronic constipation and gallstones R: bronchial asthma, febrifuge U: kidney stones K: hemorrhoids S: wounds and stab wounds X: emmenagogue	16
*Acorus calamus* L., iđirot, *Acoraceae*, IPLB44/13	Root	D: 2 (I: digestive problems, ulcers)	Decoction	D: improving appetite, digestion, against colic, diarrhea and intestinal gas R: bronchitis	2
*Allium sativum* L., beli luk, *Amarillydaceae*, IPLB28/14	Bulb	T: 2 (I: high lipid level)	Fresh	D: (I: against diarrhea and parasites) K: (I: heart pain) A: (I: malaria, against typhus and infectious diseases that are results of floods) N: (E: headache, unconsciousness) S: (E: for skin diseases, against hair loss)	1
*Allium ursinum* L*.*, sremuš, *Amarillydaceae*, IPLB26/14	Leaf Root Bulb	D: 3 (I: stimulation of digestive system) T: 1 (I: source of vitamin C)	Fresh, Tincture	/	4
*Althaea officinalis* L., beli slez, *Malvaceae*, IPLB32/14	Root Leaf Flower	R: 11 (I: cough) U: 1 (I: urinary tract infections)	Macerate, Infusion	Data only for root: R: (I: inflammation of respiratory tract) X: (E: flushing of genital tract) G: (I: against diarrhea)	12
*Anethum graveolens* L., mirođija, *Apiaceae*, IPLB21/14	Leaf	D: 1 (I: digestive complaints)	Spice	G: (I: better digestion, against flatulence) N: (I: insomnia) C: (I: hemorrhoids)	1
*Arctostaphylos uva-ursi* (L.) Spreng., medveđe grožđe, uva, *Ericaceae*, IPLB17/14	Leaf	U: 3 (I: urinary tract infections)	Infusion	U: (I: urinary infections, kidney stone)	3
*Armoracia rusticana* P.Gaertn., B.Mey. & Scherb., hren, ren, *Brassicaceae*, IPLB18/13	Root	D: 1 (E: stomach pain)	Compress	/	1
*Aronia melanocarpa* (Michx.) Elliott, aronija, *Rosaceae*, IPLB25/13	Leaf Fruit	T: 1 (I: diabetes) K: 1 (I: high blood pressure) B: 3 (I: anemia, weakened immune system)	Fresh, Juice, Infusion	/	5
*Artemisia absinthium* L., beli pelin, *Asteraceae*, IPLB17/13	Flower	D: 2 (I: gastrointestinal and liver complains) U: 1 (I: renal complaints)	Infusion	/	3
*Beta vulgaris* L., cvekla, *Amaranthaceae*, IPLB67/14	Root	B: 1 (I: anemia)	Juice, Fresh	/	1
*Brassica nigra* (L.) K. Koch., crna slačica, *Brassicaceae*, IPLB33/13	Seed	D: 1 (I: liver complaints) T: 1 (I: diabetes) L: 1 (I: rheumatism)	Decoction	R: (E: bronchitis, mild pneumonia) L: (E: rheumatism)	3
*Calendula officinalis* L., neven, *Asteraceae*, IPLB53/13	Flower, Herb	S: 8 (Е: against burns, wounds and skin complaints) D: 3 (I: digestive and liver complaints) K: 1 (E: vein problems) L: 2 (E: bone pain)	Fat based ointments, infusion, tincture	S: (E: scaly tinea, impetigo, wounds and skin ulcers, bee and wasp sting) X. (I: irregular menstrual bleeding; E: vaginal irrigation) D: (I: stomach, gut and gallbladder diseases)	14
*Camellia sinensis* L. Kuntze, zeleni čaj, *Theaceae*, IPLB1-T/13	Leaf	T: 4 (I: detoxification, antioxidant) S: 1 (E: burns) K: 1 (I: high blood pressure)	Infusion, compress	T: (I: antidote in cases of alkaloids and heavy metals poisoning) D: (I: for some stomach diseases) U: (I: mild diuretic)	6
*Capsicum annuum* L., crvena paprika, Solanaceae, IPLB73/13	Fruit	L: 1 (E: rheumatism)	Compress	D: (I: improves digestion, stomach pain) P: (I: alcoholism)	1
*Carum carvi* L., kim, *Apiaceae*, IPLB101/13	Fruit	D: 2 (I: flatulence, meteorism) U: 1 (I: diuretic)	Decoction	D: (I: improves digestion) U: (I: diuretic) X: (I: galactagogue)	3
*Cetraria islandica* L., islandski lišaj, *Parmeliaceae*, IPLB86/14	Thallus	R: 2 (I: cough)	Decoction	R: (I: cough relief) G: (I: against nausea and vomiting, tonic and stomachic, against chronic diarrhea)	2
*Chelidonium majus* L., rusopas, rusa, *Papaveraceae*, IPLB8/13	Leaf Flower	D: 1 (I: liver complaints)	Infusion, Juice	S: (E: cutaneous tuberculosis, warts removing) D: (I: bile production stimulation, gastrointestinal pain) R: (I: asthma)	1
*Cichorium intybus* L., vodopija, cikorija, gologuza, *Asteraceae*, IPLB40/14	Stem	D: 1 (I: diarrhea)	Infusion	D: (I: improving of appetite, bile ducts cleaning, inflammation of gut and stomach inflammation)	1
*Cinnamomum verum* J. Presl, cimet*, Lauraceae*, IPLB12-T/14	Bark	A: 1 (E: antiseptic)	Infusion	/	1
*Citrus x limon* (L.) Osbeck., limun, *Rutaceae*, IPLB7-T/14	Fruit Leaf	T: 2 (I: source of vitamin C) B: 1 (I: weakened immune system)	Juice	/	3
*Cornus mas* L., dren, drenjina, *Cornaceae*, IPLB14/14	Fruit	D: 1 (I: diarrhea)	Infusion	D: (I: diarrhea, treatment of gastrointestinal organs)	1
*Crataegus monogyna* Jacq., glog, *Rosaceae*, IPLB10/13	Leaf Flower Fruit	K: 8 (I: heart failure) R: 1 (I: respiratory complaints) T: 1 (I: rich source of vitamin C)	Infusion, Juice	K: (I: arrhythmia, arteriosclerosis and hypertension)	10
*Cucurbita pepo* L., tikva, *Cucurbitaceae*, IPLB114/14	Seed	Y: 1 (I: benign prostate hyperplasia)	Fresh, Mixed with honey	S: fresh or cooked pulpa for treatment of psoriasis)	1
*Cydonia oblonga* Mill., dunja, *Rosaceae*, IPLB99/13	Leaf	D: 1 (I: diarrhea)	Infusion	R: (I: antitussive agent) S: (E: for burns)	1
*Cynara scolymus* L., artičoka, *Asteraceae*, IPLB82/14	Flower	T: 1 (I: high lipid level)	Tincture	G: (I: improves digestion, choleretic and cholagogue) U: (I: mild diuretic)	1
*Daucus carota* L., šargarepa, *Apiaceae*, IPLB86/13	Root	F: 1 (I: good for improving eyesight)	Fresh	D: (I: juice is used for threadworms, against diarrhea) T: (I: juice is used for diabetes)	1
*Equisetum arvense* L., rastavić, preslica *Equisetaceae*, IPLB6/13	Herb	U: 2 (I: urinary tract ailments and infections) B: 1 (I: anemia) R: 1 (I: respiratory complaints)	Infusion	U: (I: diuretic)	4
*Ficus carica* L., smokva, *Moraceae*, IPLB116/14	Fruit	D: 1 (I: digestive complaints)	Fresh, Juice	R: (I: expectorant, as a component in tea mixtures for lungs) D: (in combination with senna leaves)	1
*Foeniculum vulgare* Mill., morač, komorač, *Apiaceae*, IPLB85/13	Fruit	D: 3 (I: digestive complaints)	Infusion, decoction, fresh	D: (I: improving of appetite, beneficial for good digestion, diminish consequences of incomplete and slow digestion)	3
*Fragaria vesca* L., jagoda, *Rosaceae*, IPLB77/14	Fruit	T: 1 (I: high lipid level)	Fresh, Juice	Rhizome: D: (I: against diarrhea, dysentery, gastrointestinal inflammations) K: (E: hemorrhoids) U: (I: diuretic)	1
*Gentiana lutea* L., lincura, *Gentianaceae*, IPLB12/12	Root	D: 3 (I: digestive problems, loss of appetite) K: 2 (E: vein complaints)	Tincture	D: (for stomach, against fever) S: (I: for wounds) R: (I: root is cooked and tea is used against cough)	5
*Geranium macrorrhizum* L., zdravac, *Geraniaceae*, IPLB53/14	Leaf Flower	D: 1 (I: digestive disorders)	Infusion	D: (I: treatment of inflamed gastrointestinal mucosa)	1
*Ginkgo biloba* L., ginko, *Ginkgoaceae*, IPLB3-T/14	Leaf	K: 1 (I: circulation disorder)	Infusion	/	1
*Hedera helix* L., bršljan, *Araliaceae*, IPLB105/13	Leaf	R: 1 (I: cough)	Syrup	S: (E: treatment of skin diseases, warts) L: (treatment of rheumatism)	1
*Hibiscus sabdariffa* L., hibiscus, *Malvaceae*, IPLB2-T/14	Flower	R: 2 (I: cold)	Infusion	/	2
*Hypericum perforatum* L., kantarion, *Hypericaceae*, IPLB9/12	Herb	S: 25 (E: against burns, wounds and skin complaints) D: 13 (I: gastrointestinal ailments) K: 3 (E: circulation disorders) R: 3 (I: cold) P: 3 (I: depression, sedative) U: 1 (I: urinary complaints)	Infusion, Oil extract	S: (E: cuts, burns, wounds) K: (E: hemorrhoids) D: (I: liver and gastric pain, diarrhea)	48
*Hyssopus officinalis* L., miloduh, *Lamiaceae*, IPLB83/14	Leaf Flower	R: 1 (I: bronchitis)	Infusion	R: (I: chronic bronchitis, asthma) G: (I: stomachic)	1
*Inula helenium* L., oman, *Asteraceae*, IPLB60/13	Leaf Flower	R: 1 (I: cough)	Infusion	R: (I: bronchitis, cough) X: (I: irregular menstrual bleeding) S: (E: dry tinea infections and itchiness) G: (I: stomachic) L: (I: rheumatism)	1
*Juniperus communis* L., kleka, *Cupressaceae*, IPLB12/13	Fruit	U: 1 (I: diuretic)	Juice, Infusion	U: (I: diuretic) R: (I: common cold, cough, asthma) X,Y: (I: gonorrhea)	1
*Laurus nobilis* L., lovor, *Lauraceae*, IPLB47/13	Leaf	R: 1 (I: cough)	Infusion	D: (I: essential oil of leaves – carminative)	1
*Lavandula angustifolia* Mill., lavanda, *Lamiaceae*, IPLB19/13	Herb	P: 1 (I: anxiety, insomnia) D: 1 (I: gastrointestinal spasms and flatulence)	Infusion	S: (E: external application, for bath, rubefacient)	2
*Linum usitatissimum* L., lan, *Linaceae*, IPLB23/14	Seed	R: 1 (I: cough)	Infusion	D: (I: treatment of inflamed mucosa of mouth, stomach and gut, gastrointestinal inflammatory diseases)	1
*Lycopodium clavatum* L., prečica, *Lycopodiaceae*, IPLB41/14	Herb	D: 1 (I: liver disorders)	Infusion	S: (E: non-infectious skin rashes and various skin diseases) U: (I: bladder and kidney diseases)	1
*Matricaria chamomilla* L., kamilica, *Asteraceae*, IPLB74/13	Herb	S: 3 (E: skin inflammation, as mouth and eyewash, for burns) D: 22 (I: digestive problems, liver disorders) R: 15 (I: cough) L: 3 (I: anxiety and insomnia)	Infusion	S: (E: burns, wounds, skin ulcers, non-infectious skin rashes) X: (E: washing genital organs) D: (E: digestion complaints) L: (I: insomnia)	43
*Melissa officinalis* L., matičnjak, *Lamiaceae*, IPLB43/13	Leaf	P: 7 (I: anxiety and insomnia, depression) N: 1 (I: migraine) L: 2 (E: rheumatism, gout) S: 1 (E: wound) D: 1 (I: digestive problems)	Infusion, Tincture, Oil extract	P: (I: hysteria) D: (I: flatulence, vomiting, diarrhea) P: (I: neurasthenia)	12
*Mentha x piperita* L., pitoma nana, *Lamiaceae*, IPLB15/13	Herb	D: 60 (I: digestive problems, meteorism, flatulence, spasms) L: 7 (I: anxiety, insomnia) N: 1 (I: headache) A: 4 (I: fever) S: 2 (E: mouthwash)	Infusion, Spice	D: (I: intestinal gas, flatulence and cramps, digestion problems as a stomachic, mild diarrhea) P: (I: anxiolytic)	74
*Mentha spicata* L., divlja nana, *Lamiaceae*, IPLB30/13	Herb	D: 1 (I: stomach disorders) R: 1 (I: cough)	Infusion	/	2
*Morinda citrifolia* L., noni, *Rubiaceae*, IPLB10-T/14	Fruit	T: 1 (I: tonic)	Tincture	/	1
*Ocimum basilicum* L., bosiljak, *Lamiaceae*, IPLB24/13	Herb, Flower, Leaf	R: 10 (I: inhalation agent, cough, asthma) D: 5 (I: gastrointestinal ailments) U: 1 (I: urinary complaints) P: 1 (I: anxiety) S: 1 (E: warts)	Infusion, Compress, Spice	P: (I: for calming of exhilarated nervous system) D: (E: against intestinal gas, flatulence, and gastrointestinal complaints, for improving of appetite) U: (I: stimulates urination, against kidney inflammation) X: (I: increasing breast milk production at nursing mothers, regulation of menstrual cycle)	18
*Origanum majorana* L., majoran, *Lamiaceae*, IPLB39/14	Flower	D: 1 (I: digestive complaints) N: 1 (I: headache)	Infusion	S: (E: skin inflammation and wound healing)	2
*Origanum vulgare* L., vranilova trava, *Lamiaceae*, IPLB27/13	Herb	U: 1 (I: urinary tract infections)	Infusion	D: (I: gastrointestinal diseases, especially diarrhea) S: (E: skin and mucosal inflammation)	1
*Petroselinum crispum* (Mill.) Fuss, peršun, *Apiaceae*, IPLB34/13	Leaf Root Seed	U: 6 (I: urinary tract infections)	Infusion, Spice	U: (I: diuretic)	6
*Pinus sylvestris* L., beli bor, *Pinaceae*, IPLB38/14	Needle	R: 1 (I: respiratory disorders)	Syrup	R: (I: chronic bronchitis) L: (E: rheumatism)	1
*Plantago major* L., ženska bokvica, *Plantaginaceae*, IPLB16/13	Leaf	R: 3 (I: cough, bronchitis) S: 2 (E: skin complaints)	Infusion, Compress	R: (I: expectorant) S: (E: skin and mucosal inflammation) D: (I: beneficial effect on gastrointestinal system, for diarrhea, stomach cramps, gastric and duodenal ulcers)	5
*Potentilla anserina* L., steža, *Rosaceae*, IPLB48/14	Stem Flower	D: 1 (I: diarrhea)	Infusion	K: (I: heart pain) D: (diarrhea)	1
*Primula veris* L., jaglika, jagorčevina, *Primulaceae*, IPLB29/13	Root Flower Leaf	R: 5 (I: cough) K: 1 (I: vein complaints)	Decoction Infusion Tincture Syrup	R: (I: cough)	6
*Prunus armeniaca* L., kajsija, *Rosaceae*, IPLB35/13	Seed	T: 1 (I: source of vitamin B17)	Fresh	/	1
*Pulmonaria officinalis* L., plućnjak *Boraginaceae*, IPLB45/13	Leaf Flower Root	R: 1 (I: respiratory disorders)	Infusion	R: (I: expectorants) U: (I: diuretic) G: (I: antidiarrhoeic agent)	1
*Punica granatum* L., nar, *Lythraceae*, IPLB52/13	Bark	D: 1 (I: diarrhea)	Infusion	D: (I: against tapeworms)	1
*Rosa canina* L., šipurak, divlja ruža, *Rosaceae*, IPLB20/13	Fruit	A: 4 (I: fever) D: 2 (I: stomach complaints) T: 2 (I: source of vitamin C) B: 1 (I: weakened immune system)	Decoction	T: (I: source of vitamin C) D: (I: astringent in gut diseases, especially at kids diarrhea) U: (I: diuretic – Cynosbati semen)	9
*Rosmarinus officinalis L.*, ruzmarin *Lamiaceae*, IPLB5/13	Leaf Flower	D: 2 (I: stomach complaints) K: 1 (I: cardiovascular complaints)	Infusion Spice	S: (E: stimulates hair growth) D: (I: carminative) X: (I: abortifacient)	3
*Rubus vulgaris* Weihe & Nees, kupina, *Rosaceae*, IPLB31/13	Leaf, Fruit	B: 1 (I: anemia)	Infusion	D: (I: chronic diarrhea) K: (I: hemmorhoides)	1
*Salix caprea* L., vrba iva, *Salicaceae*, IPLB61/14	Bark	L: 1 (I: rheumatic pain)	Infusion	–	1
*Salvia officinalis* L., žalfija, *Lamiaceae*, IPLB4/13	Herb	R: 28 (E: mouth and throat wash, cold) P: 2 (I: anxiety) D: 2 (I: for “warm” stomach) B: 1 (I: weakened immune system)	Infusion	R: (E: for mouth and throat washing in inflammatory diseases accompanied with secretion)	33
*Sambucus nigra* L., zova, *Adoxaceae*, IPLB23/13	Flower, Fruit, Leaf,	A: 12 (I: diaphoretic, fever) U: 1 (I: renal complaints) T: 2 (I: diabetes) K: 1 (I: cleaning the blood)	Infusion, Juice	A: (I: diaphoretic) D: (I: laxative) U: (I: diuretic)	16
*Sempervivum tectorum* L., čuvarkuća, *Crassulaceae*, IPLB49/14	Leaf	H: 5 (E: earache) S: 2 (E: wounds and warts)	Juice Compress, Tincture	S: (E: skin inflammation)	7
*Senna alexandrina Mill*., sena, *Fabaceae*, IPLB5-T/14	Leaf	D: 3 (I: laxative)	Infusion	/	3
*Silybum marianum* (L.) Gaertn., gujina trava, *Asteraceae*, IPLB76/14	Fruit	D: 1 (I: liver disorders)	Decoction	R: (I: asthma) A: (I: headache, tonic) D: (I: alleviates gallbladder attack, constipation) T: (I: diabetes) K: (I: hemorrhoids)	1
*Stevia rebaudiana* (Bertoni) Bertoni, stevia, *Asteraceae*, IPLB13-T/14	Leaf	T: 1 (I: sweetener instead of sugar)	Fresh	–	1
*Symphytum officinale* L., gavez, *Boraginaceae*, IPLB50/13	Flower, leaf	L: 1 (E: bone fractures, rheumatism)	Compress	L: (E: bone fracture) S: (E: for stabs, skin ulcers, old and abscess wounds) R: (I: as expectorant and for inflammation of mouth)	1
*Taraxacum campylodes* G.E.Haglund, maslačak, *Asteraceae*, IPLB70/13	Root, leaf, flower	D: 2 (I: laxative, liver disorder) T: 5 (I: diabetes, antioxidant) R: 1 (I: respiratory problems)	Infusion, Decoction, Spice	–	8
*Teucrium chamaedrys* L., podubica, *Lamiaceae*, IPLB54/14	Leaf Herb	D: 3 (I: digestive and liver complaints)	Infusion	D: (I: gallbladder problems) K: (I: hemorrhoids) S: (E: wounds) X: (E: increased vaginal discharge with no infections)	3
*Teucrium montanum* L., trava iva, *Lamiaceae*, IPLB12/14	Herb	D: 2 (I: digestive complaints)	Infusion	D: (I: gallbladder problems) K: (I: hemorrhoids) S: (E: wounds) X: (E: increased vaginal discharge with no infections)	2
*Thymus serpyllum* L., majkina dušica, *Lamiaceae*, IPLB3/13	Herb	R: 13 (I: cold, bronchitis) D: 11 (I: gastrointestinal disorders) P: 3 (I: anxiety and insomnia) X: 1 (I: gynecological complaints in women)	Infusion Syrup	D: (I: diarrhea) A: (I: infectious diseases)	28
*Thymus vulgaris* L*.*, timijan, *Lamiaceae*, IPLB2/13	Leaf	R: 1 (I: respiratory disorders)	Infusion	D: (I: intestinal parasites, diarrhea) R: (I: component of mixture for whooping cough and regular cough)	1
*Tilia cordata* Mill., lipa, *Malvaceae*, IPLB64/13	Flower	A: 16 (I: fever, diaphoretic) P: 4 (I: anxiety)	Infusion	A: (I: diaphoretic) D: (I: for diminishing pain that accompanies stomach cramps)	20
*Trifolium pratense* L., detelina, *Fabaceae*, IPLB58/14	Leaf Flower	T: 1 (I: rich source of vitamins)	Salad	/	1
*Tussilago farfara* L., podbel, *Asteraceae*, IPLB37/13	Leaf	R: 2 (I: cough, bronchitis)	Infusion	S: (E: leaves covered with lard or oil for stabs, cuts, abscessed and inflamed skin places/area) R: (I: asthma)	2
*Urtica dioica* L., kopriva, *Urticaceae*, IPLB14/13	Herb Leaf Seed	T: 7 (I: detoxification) K: 10 (I: cleaning the blood, hemorrhoids) B: 7 (I: anemia) S: 3 (E: mouthwash, loss of hair) L: 2 (E: rheumatic disorders) R: 1 (I: respiratory problems) D: 1 (I: digestive problems)	Infusion, spice, tincture, juice, seed mixed with honey	D: (I: diarrhea) X: (E: increased vaginal discharge with no infections) K: (I: for hemorrhoids, against bleeding) S: (E: stimulates hair growth, against hair loss and dandruff) L: (E: rheumatism, sciatica and neuralgia)	31
*Vaccinium myrtillus* L., borovnica, *Ericaceae*, IPLB51/13	Fruit Leaf	B: 4 (I: anemia) U: 1 (I: diuretic)	Infusion Juice	D: (I: mild antidiarrheal agent, for acute enterocolitis in adults) U: (I: diuretic) T: (I: diabetes)	5
*Vaccinium vitis-idea* L., brusnica, *Ericaceae*, IPLB8-T/14	Fruit	U: 5 (I: urinary tract infections, diuretic)	Infusion	U: (I: diuretic, treatment of kidney and bladder inflammation)	5
*Valeriana officinalis* L., odoljen, macina trava, valerijana, *Caprifoliaceae*, IPLB55/13	Leaf Flower	P: 1 (I: sedative)	Infusion, Tincture	Data only for root P: (I: calming of nervous system) D: (I: carminative) K: (I: beneficial effect on heart)	1
*Vitis vinifera* L., vinova loza, *Vitaceae*, IPLB62/14	Leaf	L: 1 (I: rheumatism)	Infusion	X: (I: regulation of menstrual cycle) Juice obtained during vine pruning – U: (I: kidney stones); F: (I: for inflamed eyes)	1
*Zea mays* L., kukuruzna svila, *Poaceae*, IPLB90/13	Stigma	U: 3 (I: urinary complaints) L: 1 (I: rheumatic pain, gout)	Infusion	U: (I: mild diuretic and calming agent for neutralization of pressure in bladder, bladder sand, chronic cystitis, nephritis) L: (I: reducing of rheumatic pain)	4
*Zingiber officinale* Roscoe, đumbir, *Zingiberaceae*, IPLB6-T/14	Root	R: 2 (I: sinus disorders) B: 2 (I: weakened immune system) K: 1 (I: circulation disorder) L: 1 (I: for better muscle mobility)	Spice, Mixed with honey	D: (I: carminative, stimulation of appetite)	6

^a^Methods of employment: I, internally; E, externally; ^●^Not reported in previously conducted ethnobotanical studies in Serbia (Jarić et al., 2007; Šavikin et al., 2013; Zlatković et al., 2014; Jarić et al., 2015); IPLB – Herbarium of the Institute for Medicinal Plants Research.

According to our study, botanical remedies comprise 85 plant species belonging to 43 families. Number of plant species cited by younger (< 40 years) and older informants (> 40 years) significantly differed in Pcinja district (p=0.01). Also, a statistically significant difference was observed among males and females on the number of plant species used for treatment of various health disorders (p=0.02). The predominant botanical families were *Lamiaceae* (30% of species), *Asteraceae* (26% of species) and *Rosaceae* (19% of species). These families include many cosmopolitan medicinal plant species that can be easily reached in the investigated area’s ecosystems. So, at least partially their wide application in traditional medicine can be attributed to their predominance in the flora of the investigated region. Around 15% of Serbian flora comprise Balkan endemic species. Still, in our study, the traditional use of endemic species was not recorded in the investigated district.

In Pčinja district species with the highest number of use reports were *Mentha piperita* (74), *Hypericum perforatum* (48), *Matricaria chamomilla* (43), *Salvia officinalis* (33), *Urtica dioica* (31), *Thymus serpyllum* (28), and *Tilia cordata* (20). Folk medicine is primarily used for healing minor diseases with some exceptions (e.g., *Viola tricolor* and *Viola odorata* for treating malignant diseases). The most frequent medicinal uses were for treating diseases of the digestive system, respiratory system and diseases of the skin and subcutaneous tissue, followed by general and unspecified diseases (such as pain, fever, malignancy and health prevention). The prominence gastrointestinal applications of medicinal plants in our study could be due to the prevalence of these ailments but also the absence of efficacious pharmaceuticals in those diverse conditions, e.g. digestive problems, diarrhea, constipation, abdominal pain, ﬂatulence, colic. Also, among the most common causes for going to the Health Centre general practitioner are respiratory diseases and that is reflected in the uses of herbal medicines.

**Table 3** indicates FIC values of the category of ailment. The level of informants’ agreement was high for most ailment categories and these points out towards the great uniformity in the selection of species used to treat diseases belonging to these ailment categories. The knowledge interchange and homogenization due to knowledge transmission can be seen as a possible explanation for the existing low number of medicinal plants used in investigated districts (Cavalli-Sforza et al., 1982). The majority of the plants (72) were reported to have 1–3 different usages and the species with the most diverse uses were *Hypericum perforatum* and *Urtica dioica.* These plant species hold important place in the Serbian traditional medicine, as well as in common beliefs. It is thus believed that on the St. Georges Day (6^th^ of May) you need to take a bath with the water in which nettle was submerged. This would contribute to general health. For the St. Johns Worth, also known in the folk medicine as Virgin Mary’s grass, there is a belief existing on the South of Serbia that it got its spots after drops of water falling from the hands of the Virgin Mary to its leaves (Čajkanović, 1985).

**Table 3:** Informants consensus factor (FIC) for different ailment categories (according to International Classification of Primary Care).

Ailment category	Abbreviation	Pčinja district			
		% of use reports	Nt	Nur	FIC
General and unspecified	**A**	7.0	5	37	0.89
Blood, blood-forming organs and immune mechanism	**B**	3.4	8	18	0.59
Endocrine/metabolic and nutritional	**T**	6.4	17	34	0.51
Psychological	**P**	4.2	8	22	0.67
Neurological	**N**	0.6	3	3	0.00
Eye	**F**	0.2	1	1	0.00
Ear	**H**	1.0	1	5	1.00
Cardiovascular	**K**	6.3	11	33	0.69
Respiratory	**R**	22.5	26	113	0.78
Digestive	**D**	33.4	37	171	0.79
Skin	**S**	9.4	12	49	0.77
Musculoskeletal	**L**	2.5	10	13	0.25
Urological	**U**	2.7	14	27	0.50
Female genital	**X**	0.2	1	1	0.00
Male genital	**Y**	0.2	1	1	0.00
Pregnancy, childbearing, family planning	**W**	0.0	0	0	–

Several different ways for preparation and administration of medicinal plants were reported. The most of all reported plants were consumed internally (79.52%), whereas 14.46% of all plants in Pčinja district were used both internally and externally and 6.02% of medicinal plants were used only externally. As far as internal consumption is concerned, in the Pčinja district, the dominant form was infusion, followed by eating fresh plant parts, using juice, tincture or decoction. Although infusions should be prepared with lighter parts of plants (leaves, herb, flowers) and decoction should be applied for extracting constituents from roots, bark, seeds, and berries, we have noticed that informants do not make such a difference. External application included infusions for gargling and rinsing, compress or fresh plant parts as cataplasm, ointments, oil extracts and tinctures. Minor preparation forms were syrup and macerate. Several medicinal plants were used as a spice.

The most used plant part was leaf (31.58%). This partially can be explained by their collection convenience. Other parts used were: flower, fruit, herb, root, seed, stem, bark, bulb, stigma, needles, and thallus.

Sixty-nine informants (64.5%) in Pčinja district confirmed that there was tradition of collecting medicinal plants in their family and 55 (51.4%) informants collect plants by themselves. In Serbia, special environmental legislation is protecting numerous medicinal and aromatic plant species. This way their rational and moderate use has been guaranteed. Some of these plant species are also mentioned by informants in investigated districts (*Alchemilla* sp., *Arctostaphylos uva-ursi*, *Betula pendula*, *Gentiana lutea*, *Juniperus communis*, *Satureja* sp., and *Vaccinium myrtillus*) (Jarić et al., 2014).

It should be noted that some of very well-known plants (e.g. *Betula pendula, Verbascum densiflorum, Aesculus hippocastanum, Humulus lupulus, Juglans regia* and *Morus nigra*) were not reported in the investigated district.

### Comparison With Previously Published Data and Novelty of Uses in the Studied Area

In order to compare traditional plant use in the investigated district with neighboring areas, four previously published ethnomedicinal studies conducted on the territory of Serbia were considered (Jarić et al., 2007; Šavikin et al., 2013; Zlatković et al., 2014; Jarić et al., 2015). Though the research areas and methods are different in these studies, similarity regarding plant use and modes of application can be expected due to the fact that these areas share similar flora, and also due to the cross-cultural knowledge exchange in the past. According to our results, considering 86 plant species recorded in Pčinja district, 23 of them were not mentioned in four cited studies from neighboring areas. Several of these species are not characteristic for the Serbian flora: *Camellia sinensis*, *Senna alexandrina*, *Cinnamomum verum* and *Morinda citrifolia*. Species mentioned in investigated area, as well as in areas used for comparison are: *Achillea millefolium*, *Allium ursinum*, *Althaea officinalis*, *Cichorium intybus*, *Equisetum arvense*, *Hypericum perforatum*, *Matricaria chamomilla*, *Mellisa officinalis*, *Plantago major*, *Primula veris*, *Sambucus nigra*, *Sempervivum tectorium*, and *Urtica dioica*. The similarity between data on medicinal plants obtained in current as well as in studies previously conducted in surrounding regions is presented in **Table 4**. Similarly, to Zlatibor district number of cited plant species per informant is lower compared to surrounding areas (Kopaonik, Suva planina, and Rtanj), suggesting the disappearance of ethnobotanical practice.

**Table 4:** Comparison between the medicinal plant uses in Pčinja district and those previously recorded in ethnobotanical studies conducted in surrounding regions.

Area	Year(s) when the field studies were conducted	Number of study participants	Number of recorded plant species	Number of plant species cited per informant	% of plant species also quoted in Pčinja district
Zlatibor district (Šavikin et al., 2013)	2011	220	69	0.31	68.12
Kopaonik (Jarić et al., 2007)	2002–2005	60	83	1.38	48.19
Suva planina (Jarić et al., 2015)	2012–2014	66	128	1.94	39.06
Rtanj (Zlatković et al., 2014)	2011–2012	37	45	1.27	40.00
Pčinja district	2015	107	86	0.80	–

Also during this research comparison has been made with the knowledge about traditional medicine that had been recorded by Dr. Jovan Tucakov. In the book called Herbal therapy he sees traditional medicine as a “treasure chest”. According to this comparison 17 plant species recorded during ethnobotanical investigation in Pčinja district have not been mentioned in the Jovan Tucakovs’ book. However, for plant species mentioned by Tucakov much more diverse application is elaborated compared to modern-day accounts from Pčinja district. Examples for this are the following species: *Inula helenium*, *Juniperus communis*, *Pulmonaria officinalis*, *Symphytum officinale*, *Teucrium chamaedrys*, *Chelidonium majus* (**Table 2**). In this respect, Tucakov says for the *Teucrium chamaedrys* that it is one of the most favorable folk medicine of Serbs, especially in mountainous regions. There is a practice of making wine from *T. chamaedrys* (200 g is poured with liter of red wine and is left to stay for 8 days) which is used by the people as the cure against weaknesses, anemia and for wound washing. Another plant that Tucakov describes as one of the most favorite plants of the Serbian people is garlic. For this species, he says there are only a few local plants which acquired so much importance and in which people have such firm confidence as is the case with garlic. Consequently, Tucakov lists several examples recorded among Serbian population:„it is useful to eat garlic on a daily basis in order to fight cholera and typhus”; there is no such diarrhoea or heart pain which can not be cured with garlic and oak peel” (Tucakov, 1997).

Also, for different plant species, various plant organisms are recorded. For example, Tucakov emphasizes the use of *Fragaria vesca* rhizome in traditional medicine, while respondents in the Pčinja region use strawberry fruit for medicinal purposes. Also, while Tucakov states the application of the valerian root as a sedative, the modern-day inhabitants of the Pčinja district region use valerian leaf and flower. According to our knowledge, there are no data from investigations that could justify such an application.

Another conclusion which can be made from this comparison is that half a century ago a greater number of plant species have been used for so-called female diseases. Tucakov (1997) names as much as twelve plant species (*Achillea millefolium*, *Althaea officinalis*, *Calendula officinalis*, *Carum carvi*, *Inula helenium*, *Juniperus communis*, *Matricaria chamomilla*, *Ocimum basilicum*, *Rosmarinus officinalis*, *Teucrium chamaedrys*, *Teucrium montanum*, *Urtica dioica*, *Vitis vinifera*) that can be used for this group of diseases. One of the reasons for such a situation can be found in the fact that Serbia was in that time overwhelmingly peasants’ society and visits to the doctor were not part of their culture. Also, the number of doctors was scarce.

In order to highlight new or rare uses of medicinal plants, collected data were compared with regional, national and global uses of plants. Most of the recorded species in both districts are traditionally well known, and after comparison with literature - PDR (Physicians Desk References) for Herbal Medicines (2000) some of them have shown new uses. Few examples are discussed below:

- Juice of *Sempervivum tectorum* has been used in earache treatment. This has also previously reported in traditional medicine of other regions in Serbia (Jarić et al., 2007; Šavikin et al., 2013). Also, Stojković et al. (2015) showed that the juice possesses antimicrobial activity towards clinical isolates of bacteria linked to otitis. Antimicrobial activity was tested using a microdilution assay on bacteria (*Proteus mirabilis*, *Staphylococcus aureus*, and *Pseudomonas aeruginosa*) isolated from ear swabs of the patients suffering from the ear pain. Reference strain of *P. aeruginosa* (ATCC 27853) was the most sensitive to the influence of *S. tectorum* juice, with MIC of 0.153 mg/mL and MBC 0.290 mg/mL. In the same investigation quorum sensing functions in *Pseudomonas aeruginosa* were effectively controlled with juice obtained from leaf of *Sempervivum tectorum*.
- Corn silk is used for the treatment of gout and rheumatic pain. As a well-known diuretic corn silk (*Zea mays*) stimulates elimination of toxins and uric acid from the body, which can be a possible explanation for relieving gout, edema and arthritis. Nile and Park (2014) showed that polyphenolic compounds isolated from maize (peonidin-3-glucoside, 3’-methoxyhirsutrin, vanillic acid and ferulic acid) exhibited significant inhibition of xanthine oxidase *in vitro* in spectrophotometric assay and formation of hydroperoxide (8.2–8.7 µM) compared to standard compound allopurinol (7.5 µM).
- Gentian root is used for vein complaints. Previously, researchers showed that gentian extract as well as its compound isovitexin possess significant anti-atherogenic properties (Kesavan et al., 2016). Diet supplemented with 2% gentian root powder prevented atheroma formation in streptozotocin induced diabetic rats after 12 weeks of application. In the same study authors recorded a significant decrease of the circulating levels of total cholesterol as well as lipid deposition on the aortic arch.

## Conclusions

The current study represents a useful documentation which can contribute to preserving ethnobotanical knowledge in South-Eastern Serbia. After comparing our results with the ones collected half a century ago by Dr. Jovan Tucakov, we can conclude that plant species mentioned in our investigation previously had a much wider spectrum of application. Also, information obtained during this investigation has shown lower number of used plant species when compared to other studies carried out in neighboring regions. For some well-known species new applications have been recorded what can present a good starting point for new investigations.

## Data Availability Statement

The raw data supporting the conclusions of this article will be made available by the authors, without undue reservation.

## Ethics Statement

Ethical review and approval was not required for the study on human participants in accordance with the local legislation and institutional requirements. The patients/participants provided their written informed consent to participate in this study.

## Author Contributions

JŽ conceived and designed the research, was enrolled in all the analyses and prepared the draft version of the manuscript. MI and AI performed data collection, curation, and methodology. KŠ and GZ were enrolled in the analysis of data and edited the final manuscript. DS performed data collection, interpreted results, and edited the final draft of the manuscript.

## Funding

This work has been supported by Ministry of Education, Science and Technological Development of Republic of Serbia (451-03-68/2020-14/200007 and 451-03-68/2020-14/ 200003).

## Conflict of Interest

The authors declare that the research was conducted in the absence of any commercial or financial relationships that could be construed as a potential conflict of interest.
